# Age-Related Differences in Strategy in the Hand Mental Rotation Task

**DOI:** 10.3389/fnhum.2021.615584

**Published:** 2021-03-10

**Authors:** Izumi Nagashima, Kotaro Takeda, Yusuke Harada, Hideki Mochizuki, Nobuaki Shimoda

**Affiliations:** ^1^Department of Occupational Therapy, Faculty of Health Sciences, Kyorin University, Mitaka, Japan; ^2^Faculty of Rehabilitation, School of Healthcare, Fujita Health University, Toyoake, Japan; ^3^Department of Rehabilitation, Faculty of Health Sciences, Tokyo Kasei University, Sayama, Japan

**Keywords:** mental rotation, motor imagery, visual imagery, performance strategy, multigeneration, inverse efficiency score

## Abstract

Mental imagery of movement is a potentially valuable rehabilitation task, but its therapeutic efficacy may depend on the specific cognitive strategy employed. Individuals use two main strategies to perform the hand mental rotation task (HMRT), which involves determining whether a visual image depicts a left or right hand. One is the motor imagery (MI) strategy, which involves mentally simulating one’s own hand movements. In this case, task performance as measured by response time (RT) is subject to a medial–lateral effect wherein the RT is reduced when the fingertips are directed medially, presumably as the actual motion would be easier. The other strategy is to employ visual imagery (VI), which involves mentally rotating the picture and is not subject to this medial–lateral effect. The rehabilitative benefits of the HMRT are thought to depend on the MI strategy (mental practice), so it is essential to examine the effects of individual factors such as age, image perspective (e.g., palm or back of the hand), and innate ability (as indicated by baseline RT) on the strategy adopted. When presented with pictures of the palm, all subjects in the current study used the MI strategy, regardless of age and ability. In contrast, when subjects were presented with pictures of the back of the hand, the VI strategy predominated among the young age group regardless of performance, while the strategy used by middle-age and elderly groups depended on performance ability. In the middle-age and elderly groups, the VI approach predominated in those with high performance skill, whereas the MI strategy predominated among those with low performance skill. Thus, higher-skill middle-aged and elderly individuals may not necessarily form a motion image during the HMRT, potentially limiting rehabilitation efficacy.

## Introduction

Motor imagery (MI) is a cognitive process of mental simulation of actions without any concomitant bodily movement and is classified into intentionally and unintentionally generated motor imageries (explicit/implicit) (Hanakawa, 2016). It is further distinguished into a first- or third-person perspective (Mulder, 2007). Hand mental rotation task (HMRT) in which subjects are required to judge whether an image represents the right or left hand, elicits the first-person MI implicitly. The HMRT is also widely used to assess cognitive function, induce regional brain activity in neuroimaging studies, and more recently as a potential tool for rehabilitation (Moseley, 2004; Harada et al., 2016; Shibui et al., 2016; Polli et al., 2017; Dilek et al., 2018). In most instances, baseline skill or acquired performance is measured by response time (RT) (Sekiyama, 1982; Parsons, 1987; Saimpont et al., 2009; Takeda et al., 2010; Mochizuki et al., 2019). One plausible cognitive approach to task performance is the MI, in which the subject forms a moving mental image of their own hand and superimposes it onto the presented picture. The RT profile of this strategy is characterized by the medial–lateral effect (De Simone et al., 2013), whereby the RT is shorter when the tip of the middle finger faces the medial side of the body, probably because the image is easier to superimpose compared to the condition where the middle finger faces the lateral side of the body. This medial–lateral effect has been confirmed across all ages for the palm picture-based HMRT (ter Horst et al., 2010; Bläsing et al., 2013; Zapparoli et al., 2014, 2016; Conson et al., 2017; Nagashima et al., 2017, 2019). An alternative strategy is to use visual imagery (VI), and young individuals reportedly use this VI strategy when presented with pictures of the back of the hand (ter Horst et al., 2010; Bläsing et al., 2013; Zapparoli et al., 2014, 2016). In such cases, the RT profile is not influenced by the medial–lateral effect but rather by the rotation angle of the hand picture.

For clinical application, it is believed that patients must perform the HMRT using MI, in which case the RT profile should show the medial–lateral effect. However, patients with hemiparetic cerebral palsy used the VI strategy to perform the HMRT for both images of the palm and back of the hand (Crajé et al., 2010). In contrast, patients with hemiparesis after stroke did show the medial–lateral effect (Harada et al., 2016). In addition, we found that elderly subjects adopted different strategies according to task performance (Nagashima et al., 2019), indicating that the strategy used may differ depending on baseline ability (as determined by RT) even within the same age group. Therefore, to tailor rehabilitation programs for optimal therapeutic efficacy, it is necessary to investigate how age influences HMRT performance and strategy.

While there are clear differences in cognitive abilities between very young adults and the elderly, changes during middle-age are more variable, with one study concluding that the cognitive functions underlying HMRT performance are stable from adulthood to approximately 60 years of age (Plassman et al., 1995) and another finding a slow decline after age 30 (Salthouse, 2009b) with a more accelerated decline starting at around 60 years. Wang et al. (2020) reported a shorter RT in people aged 30–39 years compared to elderly subjects aged 70–81 years, but they did not examine intermediate age groups or differences in performance strategy. Changes in HMRT performance and strategy during middle-age have important implications for the design of rehabilitation regimens as the incidence of stroke increases progressively during this period (Feigin et al., 2017).

The present study was designed to examine if the RT profile in middle-age resembles the young profile or an elderly profile more dependent on baseline skill and image perspective. To this end, we recruited over 300 participants of a broad age range and compared predominant HMRT strategy (MI or VI) among the young group (under 30 years old), middle-aged group (30–59 years old), and elderly group (over 60 years).

## Materials and Methods

### Participants

The study population consisted of 307 right-handed local volunteers who were divided into three groups based on age: a young group (15–29 years; 23 men, 38 women), a middle-age group (30–59 years; 50 men, 58 women), and an elderly group (60–88 years; 59 men, 79 women). A self-administered questionnaire was used to confirm the absence of central nervous system diseases, mental disorders, upper extremity dysfunction, and visual impairment at the time of measurement. The Edinburgh Handedness Inventory (Oldfield, 1971) was used to assess hand dominance, and participants with a laterality quotient less than 65 were excluded (three in the young group and three in the middle-age group). The participants received a verbal or written explanation of the purpose and methods of this study before providing written consent. This study was conducted with the approval of the Ethical Review Committee of Kyorin University (approval number: 27–32) and in accordance with the Declaration of Helsinki on human subjects in research. For the elderly group, data were also extracted from 106 participants of our previous study since the experimental procedure was identical (Nagashima et al., 2019).

### Experimental Procedure

The participants were seated in a quiet room on a chair in front of a 15.6-inch laptop computer (Latitude 15 3000 Series; Dell-Japan Corp., Kawasaki, Japan) connected to an external keyboard (TK-FCP026BK; ELECOM Corp., Osaka, Japan). The participants were allowed to adjust the heights of the chair and desk to obtain a comfortable seating posture. A chinrest was also employed to maintain head position and a uniform 60-cm distance to the center of the laptop screen. The participants were then asked to place their left index finger on the F key and their right index finger on the J key of the external keyboard, and their hands were then covered.

**FIGURE 1:**
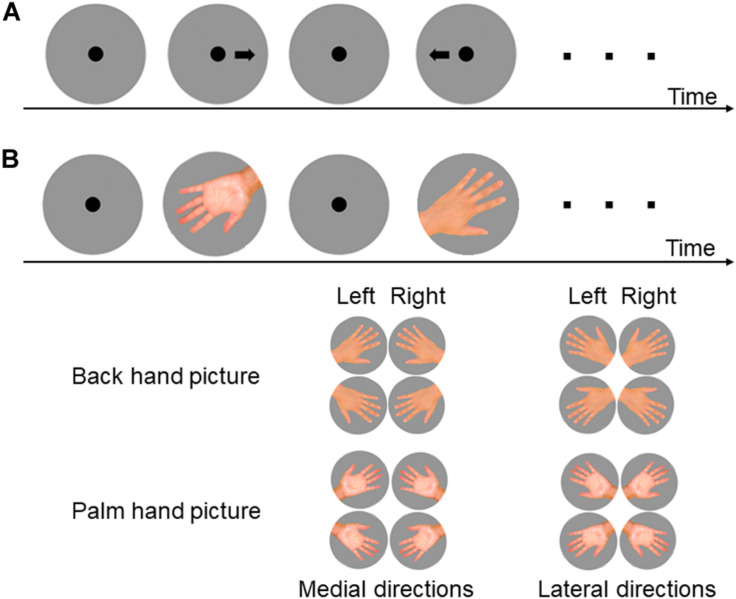
Experimental procedures and pictures presented for the left–right arrow task **(A)** and hand mental rotation task **(B)**. **(A)** Following presentation of a fixation point for 1.5 s, a picture of an arrow pointing to the left or right was presented and subjects were simply required to indicate the correct direction. **(B)** In the hand mental rotation task (HMRT), a picture of a hand back (back-of-hand condition) or palm was presented with index finger point medial or lateral. Subjects were required to determine whether the hand was the left or the right based on visual or motor imagery.

First, subjects performed an arrow-based left–right selection task, a practice run of the HMRT using six pictures of hands, and then the actual task. In the arrow-based left–right selection task, a left or right arrow was presented 15 times. In the HMRT, the pictures depicted hands with fingers fully extended and splayed, with the middle finger rotated clockwise in increments of 60° from the vertical to yield six orientations. A total of 24 hand pictures (left and right hands × palm and back-of-hand perspectives × 6 orientations) with four repetitions each were presented in random order, for 96 trials in total (Figure 1). The participants were asked to judge the direction of the arrow or the laterality of the hand as quickly and accurately as possible by pressing the F key to indicate a left arrow or hand and the J key to indicate a right arrow or hand. The arrow or picture of the hand disappeared after the response, and a fixation point measuring 3 cm in diameter was presented for 1.5 s before the next picture was displayed. The time between the presentation of the picture and the key press (RT) and response accuracy (correct or incorrect) were recorded. The picture presentations and measurements were performed using E-prime 2.0 (Psychology Software Tools, Inc., Pittsburgh, PA, United States).

### Response Accuracy Rate and Response Time

The response accuracy rate (proportion of correct answers) and mean RT were calculated for each participant in the arrow-based left–right selection task. The RTs from the 8th to the 15th arrow trials was averaged and used as an estimate of the motor response generation time. In the HMRT, the rate of correct answers was calculated and an average ΔRT calculated by subtracting the motor response generation time from the average RT for correct answers. Participants who responded incorrectly on all four presentations of the same picture were eliminated from the analysis. The experimental procedures, methods for calculating the correct answer rate, and definition of RT were the same as in our previous study (Nagashima et al., 2019).

In the present study, an inverse efficiency score (IES) (Townsend and Ashby, 1978; Deconinck et al., 2019) was also calculated by further dividing the ΔRT by the rate of correct answers. The mean IES and standard deviation (SD) were calculated for each hand picture type within each age group. Participants achieving an IES within ± 3SD of the mean for all hand pictures were included in the analysis and divided into three performance groups within each age group (Short, Medium, and Long groups in order of increasing IES). Finally, the mean IES was calculated for the medial and lateral hand pictures (Figure 1).

### Statistical Analysis

To assess age-related differences in task performance, the chi-square test among age groups was performed on participants who responded incorrectly all four times to the same hand picture.

A two-way analysis of variance (ANOVA) was conducted to examine age-related differences in the accuracy (correct response rate) of the simple left–right judgment, with age as a within subject factor and arrow direction (left vs. right) as a between subject factor. A three-way ANOVA was also conducted for the motor response generation time (velocity of the simple left–right judgment), with arrow direction (left or right) as a within subject factor, and both age range (young, middle-age, or elderly) and performance (Short, Medium, and Long) as between subject factors.

To examine differences in HMRT performance strategy, a three-way ANOVA was performed on the mean IES values for the palm and back-of-hand picture conditions, with hand direction (medial or lateral) as a within subject factor and both age and performance group as between subject factors.

In all cases, the significance level was set to 5%. We applied Bonferroni correction for *post hoc* multiple comparisons tests (paired *t*-test within subjects and Welch’s *t*-test between subjects) when significant main effects or interactions were obtained by ANOVA. All statistical analyses were conducted using SPSS Statistics (ver. 24.0; IBM Corporation, Armonk, NY, United States).

## Results

Of the 301 subjects enrolled in the study, we excluded 54 who judged the same hand picture incorrectly all four times it was presented (2 of 56 in the young group, 10 of 95 in the middle-age group, and 42 of 96 in the elderly group). A significantly greater proportion of the elderly group was excluded from the analysis due to this response inaccuracy (*p* < 0.001). In addition, 63 participants (14 in the young, 27 in the middle-age, and 22 in the elderly group) were also excluded from the analysis because their mean IES values (used as a performance metric) were outside ± 3SD from the overall age group mean. Hence, data from 184 subjects were analyzed in the present study (Table 1).

**TABLE 1:** Characteristics of the performance groups based on age.

Age group, Range (yrs.) M (SD) (yrs.)	Young, 15–29 21.5 (4.14)			Middle-age, 30–59 42.9 (8.23)			Elderly, 60–88 73.2 (7.34)		
Performance Group (*n*)	Short (14)	Medium (14)	Long (14)	Short (23)	Medium (22)	Long (23)	Short (25)	Medium (24)	Long (25)
IES range (s)	−0.78	0.78–0.89	0.89–	−0.87	0.87–1.11	1.11−	−1.30	1.30–1.86	1.86−
Men/Women	7/7	6/8	3/11	14/9	8/14	10/13	16/9	10/14	12/13
Age M (SD) (yrs.)	21.9 (2.56)	22.5 (4.26)	19.9 (5.05)	43.1 (8.03)	44.0 (9.07)	41.5 (7.75)	73.8 (7.22)	73.3 (7.69)	72.6 (7.37)
LQ M (SD)	97.9 (8.02)	95.3 (10.2)	93.5 (10.1)	95.1 (9.68)	97.3 (7.03)	94.8 (8.99)	97.6 (6.63)	96.7 (7.61)	96.8 (6.33)
Education M (SD) (yrs.)	14.6 (1.70)	14.5 (2.31)	12.5 (2.98)	16.8 (3.69)	15.8 (3.54)	16.2 (3.38)	12.6 (2.52)	12.6 (2.47)	13.3 (2.39)
RT for arrow M (SD) (s)	0.28 (0.03)	0.29 (0.05)	0.26 (0.03)	0.28 (0.03)	0.30 (0.05)	0.28 (0.03)	0.33 (0.05)	0.38 (0.09)	0.38 (0.09)
HMRT accuracy M (SD) (%)	96.8 (2.18)	96.5 (3.85)	95.0 (3.68)	95.7 (3.04)	95.7 (4.34)	95.1 (3.85)	94.3 (3.58)	90.6 (5.27)	90.4 (4.37)
ΔRT for HMRT M (SD) (s)	0.61 (0.11)	0.79 (0.06)	1.01 (0.21)	0.60 (0.12)	0.91 (0.12)	1.25 (0.25)	0.81 (0.20)	1.26 (0.18)	1.92 (0.46)

*There were no significant differences in sex ratio, laterality quotient (LQ) in the Edinburgh Handedness Inventory, and education among performance within each age group. Mean age also did not differ among performance groups within each age group. HMRT, hand mental rotation task; IES, inverse efficiency score; M, mean; RT, response time; SD, standard deviation; yrs., years.*

A two-way ANOVA for response accuracy (correct answer rate) in the arrow-based left–right selection task revealed a main effect of age [*F*(2, 181) = 6.26, *p* = 0.002], with significantly higher accuracy in the young group (100%) compared to both the middle-age group and elderly group [vs. middle-age (99.7%), *t*(67) = 2.546, Bonferroni corrected *p* = 0.013; vs. elderly (99.1%), *t*(73) = 3.744, Bonferroni corrected *p* < 0.001]. Three-way ANOVA for the motor response generation time revealed a significant main effect of age [*F*(2, 175) = 47.76, *p* < 0.001] and *post hoc* analysis revealed a significantly longer mean RT in the elderly group (0.40 s) compared to the young and middle-aged groups [vs. young (0.29 s), *t*(108.428) = 7.603, Bonferroni corrected *p* < 0.001; vs. middle-age (0.31 s), *t*(107.037) = 6.578, Bonferroni corrected *p* < 0.001]. Although the three-way ANOVA showed a significant interaction of performance and arrow direction [*F*(2, 175) = 3.88, *p* = 0.023], the *post hoc* multiple comparisons did not indicate any significant difference between any performance groups at any arrow direction.

**FIGURE 2:**
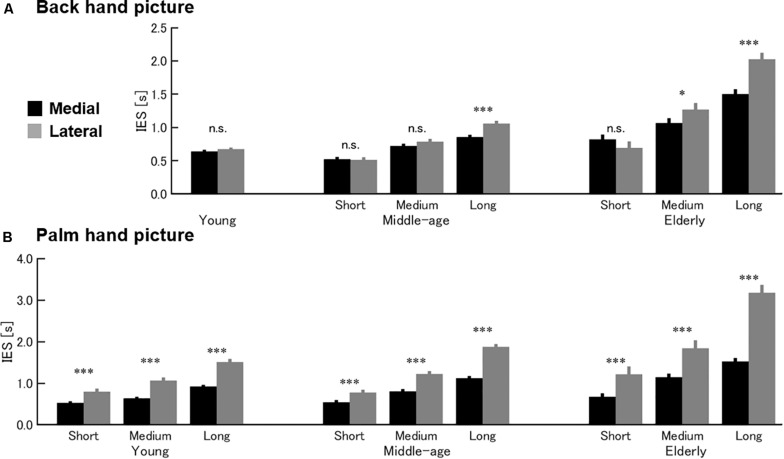
Effects of age on HMRT strategy. Mean and standard error of the inverse efficiency score (IES) for the back-of-hand condition **(A)** and palm picture condition **(B)** of the HMRT. **(A)** In the back-hand condition, a medial–lateral effect was observed in the Long performance (lower-skill) group within middle-age [*t*(22) = 3.461, *p* = 0.002] and in the Medium and Long groups within elderly age [Medium, *t*(23) = 2.279, *p* = 0.032; Long, *t*(24) = 3.751, and *p* = 0.001] groups. Thus, the entire young group [*t*(41) = 1.288, *p* = 0.205] and higher-level performance groups (Short, Medium) within the older age groups [middle-age, Short, *t*(22) = 0.621, *p* = 0.541, Medium, *t*(21) = 1.422, *p* = 0.17; elderly, Short, *t*(24) = 1.722, *p* = 0.098] appeared to use the VI strategy in this task condition. **(B)** In contrast, all performance groups within all age groups demonstrated a medial–lateral effect in the palm picture condition, indicating use of the MI strategy. young, Short, *t*(13) = 6.673, *p* < 0.001, Medium, *t*(13) = 5.345, *p* < 0.001, Long, *t*(13) = 9.032, *p* < 0.001; middle-age, Short, *t*(22) = 5.718, *p* < 0.001, Medium, *t*(21) = 5.837, *p* < 0.001, Long, *t*(22) = 7.976, *p* < 0.001; elderly, Short, *t*(24) = 5.513, *p* < 0.001, Medium, *t*(23) = 5.211, *p* < 0.001, Long, *t*(24) = 6.521, *p* < 0.001). *p < 0.05; ***p < 0.001; n.s., not significant.

Figure 2A presents the results for the HMRT back-of-hand picture condition. Three-way ANOVA revealed a significant second-order interaction [*F*(4, 175) = 4.07, *p* = 0.004]. In the young group, there was no main effect of medial–lateral direction [*F*(39, 1) = 1.59, *p* = 0.215] or an interaction between performance and medial–lateral direction [*F*(39, 2) = 0.14, *p* = 0.867]. In the middle-age and elderly groups, however, there was an interaction between performance group and medial–lateral direction [middle-age: *F*(65, 2) = 6.13, *p* = 0.004; elderly: *F*(71, 2) = 9.72, *p* < 0.001]. Within middle-age and elderly age groups, a medial–lateral effect (shorter IES values for hand images with medial direction than lateral direction) was found in the Long performance group of middle-age and in the Medium and Long performance groups of elderly but not in the higher performance groups. Thus, all performance groups within the young age group used the VI strategy (no medial–lateral effect). In contrast, middle-aged and elderly subjects with low basal task skill (longer IES) in the back-of-hand picture task condition appeared to use the MI strategy as evidenced by a significant medial–lateral effect.

Figure 2B presents the results for the HMRT palm picture condition. In this case, three-way ANOVA also revealed a significant second-order interaction [*F*(4, 175) = 3.24, *p* = 0.014]. In all age groups, there was a significant interaction between performance group and medial–lateral direction [young: *F*(39, 2) = 6.24, *p* = 0.004; middle-age: *F*(65, 2) = 13.44, *p* < 0.001; elderly: *F*(71, 2) = 11.85, *p* < 0.001]. Further, the medial–lateral effect was found in all performance groups within all age groups. Therefore, in contrast to the back-of-hand picture task condition, all age groups appeared to use the MI strategy for the palm picture condition.

## Discussion

The present study revealed a significant variation in HMRT strategy profile among subjects of different ages and performance levels (baseline skill). In the young group, there were no differences in strategy among participants with high, intermediate, and low skill levels (Short, Medium, and Long IES groups, respectively) for both the back-of-hand picture condition, in which the VI strategy was always dominant, and for the palm picture condition, in which the MI strategy was always dominant. Among subjects in the middle-age and elderly groups, however, strategy differed according to performance level specifically in the back-of-hand picture condition, with the MI strategy predominant in the lower performance skill groups, whereas the VI strategy was dominant in the higher performance skill groups. Conversely, the MI strategy was dominant under the palm picture condition, irrespective of performance, similar to the young group. Thus, subjects older than 30 years (the cut-off dividing young from middle-aged groups) demonstrated a performance strategy pattern similar to that of elderly participants and distinct from younger participants under the back-of-hand condition.

Our results for younger individuals corroborate the findings of previous studies (ter Horst et al., 2010; Bläsing et al., 2013; Zapparoli et al., 2014, 2016; Conson et al., 2017). For instance, ter Horst et al. (2010) compared the RT for three different tasks of increasing expected difficulty due to the MI complexity involved: (1) back-of-hand pictures rotated around a sagittal axis; (2) palm and back-of-hand pictures rotated around a sagittal axis; and (3) palm and back-of-hand pictures rotated around the sagittal and horizontal axes. Consistent with the current result, RT was longer in the lateral direction than the medial direction under the palm picture condition (medial–lateral effect suggesting MI strategy) but not the back-of-hand condition (no medial–lateral effect suggesting VI strategy). Further, their study also demonstrated longer RTs for more complex picture rotations requiring more complex MI, consistent with several studies reporting that changing upper extremity position increased RT (de Lange et al., 2006; Ionta et al., 2007; Ionta and Blanke, 2009). In the present study, the subjects performed the tasks with their hands concealed from view. Had a subject attempted to superimpose their hand onto the presented palm picture from this position, flexion of the shoulder joint and supination of the elbow would have been required. It may be that the MI strategy is dominant for palm pictures due to greater MI complexity compared to the back-of-hand picture condition. It also follows that, regardless of age and skill, practice based on the presentation of pictures, including palms, could better promote MI when applying HMRT during rehabilitation.

Under the back-of-hand task condition, the VI strategy was dominant among subjects in the young group and among those with higher performance skill in the middle-age and elderly groups. Conversely, the medial–lateral effect, which is characteristic of the MI strategy, was found only among middle-age and elderly subjects with lower performance skill. An IES obtained by dividing ΔRT by the rate of correct answers was used as an index of performance, and results were similar to those of our previous study that considered only ΔRT (Nagashima et al., 2019). The IES was introduced in this study to address whether there is a trade-off between response speed (RT) and accuracy (correct response rate) (Townsend and Ashby, 1978; Deconinck et al., 2019). As accuracy was not necessarily reduced by faster response as previously report (Deconinck et al., 2019; Nagashima et al., 2019), we speculated that the trend in performance would become clearer using the IES. Indeed, with further widening of the age range, the present study demonstrated that only middle-age and elderly individuals with lower performance skill used the MI strategy in the back-of-hand condition, while those of high-performance skill used the VI strategy similar to young participants. The use of VI may be explained by enhanced visuospatial transformation ability for this specific stimulus, as the hand back is viewed with greater regularity than the palms during common tasks, including typing on a keyboard (Zapparoli et al., 2014). On the other hand, middle-aged and elderly participants with lower performance skill may have used the MI strategy for the back-of-hand condition due to an age-dependent decline in visuospatial cognition and processing capabilities (Rabbitt et al., 2004; Salthouse, 2009a). Subjects with reduced visual information processing ability may also experience a decline in VI ability and thus use MI under certain conditions. This notion is supported by the lack of significant differences among the performance groups in motor response generation time for the simple left–right arrow judgment task. Conversely, in motor disorders such as Parkinson’s disease, visual information may be utilized during the HMRT to compensate for decreased MI ability associated with impaired upper limb movement (Helmich et al., 2007). Collectively, these findings suggest that MI or VI can be used to compensate for a deficit in the other strategy during the HMRT. However, a direct comparison of visual information processing ability among the subjects is necessary in future studies to test these notions. Nonetheless, the difference in strategy adopted among middle-aged and elderly individuals suggests that the HMRT could be used to measure the decline in VI or MI with age.

The first-person perspective MI needs to be induced by the HMRT for the clinical practice in rehabilitation. While the HMRT elicits the implicit and first-person perspective MI generally, the present study suggested a complementary relationship of MI and VI for the back-of-hand condition in middle-aged and elderly individuals. Therefore, an explicit MI may be more effective for the patients who are suspected of having a decline in MI ability that comes with age. For example, Gandola et al. (2019) showed a useful impact of explicit and first-person MI on motor performance and pain in relatively old patients (over 60 in average age) with rhizarthrosis. On the other hand, however, patients with cognitive dysfunction due to central nervous system disorders (e.g., stroke) often feel difficult to simulate the movements of their body even if the motion is explicitly instructed and may reduce their motivation to participate in the rehabilitation. The HMRT, which can induce MI simply by discriminating between the left and right hands, especially using palm hand pictures, may have an advantage for such patients. In conclusion for the clinical application, although it is necessary to consider the cognitive function of the patients, instructing the strategy for the HMRT, which potentially induces the implicit MI, becomes explicit, may boost the MI strategy, and consequently provide more effective rehabilitation. A further multifaceted study that includes aspects of brain function should be required to clarify these issues as discussed in Gandola et al. (2019).

In aging research, it is important to distinguish between the effects of aging *per se* and era-specific environmental factors such as education, culture, and currently available technology (Salthouse, 2019). In the present study, those in the young group showed no variation in performance strategy regardless of performance status (skill level), but it is unclear if this is an effect of age alone or due to environmental factors unique to this generation, such as frequent home game console use. Children born in the late 1980s, when home game consoles gained popularity, likely became familiar with imagining the movements of the objects displayed on computer screens at an early age (Kim et al., 2011) and so may favor the VI strategy. Indeed, gaming experience reportedly improves VI ability and visual information processing (Green and Bavelier, 2006; Achtman et al., 2008) as well as visual working memory (Blacker et al., 2014). In other words, it is unclear if the HMRT strategy pattern of this younger group will resemble that of middle-age and elderly subjects in the future, a question that warrant continued longitudinal study of this population. Furthermore, an evaluation of gaming habits among age groups may be an important factor to control for subsequent comparative studies.

## Data Availability Statement

The raw data supporting the conclusions of this article will be made available by the authors, without undue reservation.

## Ethics Statement

The studies involving human participants were reviewed and approved by Ethical Review Committee of Kyorin University. Written informed consent to participate in this study was provided by the participants or their legal guardian/next of kin.

## Author Contributions

IN and YH performed the experiments. IN, YH, and HM analyzed the data. IN, KT, and NS interpreted the results. IN drafted the manuscript. KT and NS reviewed and edited the manuscript. All authors conceived and designed the experiments. All authors contributed to the article and approved the submitted version.

## Conflict of Interest

The authors declare that the research was conducted in the absence of any commercial or financial relationships that could be construed as a potential conflict of interest.
